# Prevalence and determinants of undernutrition in the urban slums of Belagavi: a cross-sectional study among young children

**DOI:** 10.3389/fped.2025.1559692

**Published:** 2025-05-14

**Authors:** Deshna Oswal, Mubashir Angolkar, N. S. Mahantashetti, Mayank Singh, Shivani Haritay, Madan Godbole

**Affiliations:** ^1^ Department of Public Health, J.N. Medical College, KLE Academy of Higher Education & Research, Belagavi, India; ^2^ Department of Paediatrics, J.N. Medical College, KLE Academy of Higher Education & Research, Belagavi, India; ^3^ Department of Epidemiology and Biostatistics, KLE Academy of Higher Education & Research, Belagavi, India; ^4^ Food and Micro-Nutrient Analysis Laboratory, KLE Academy of Higher Education & Research, Belagavi, India

**Keywords:** undernutrition, urban slums, determinants, young children, undernutrition in India, stunting, wasting, maternal factors

## Abstract

**Introduction:**

Rapid urbanization in low- and middle-income countries has led to the expansion of slums, where children face a heightened risk of undernutrition. This study aimed to determine the prevalence of undernutrition and its determinants among children residing in the urban slums of Belagavi, Karnataka.

**Methods:**

The anthropometric measurements, clinical signs, demographic information, and dietary history of children aged 9–36 months from urban slums were assessed. The chi-square test, bivariate analysis, and multivariable logistic regression were used to identify the risk factors at the child, maternal, and household levels for undernutrition.

**Results:**

The prevalence of stunting, wasting, and underweight among children aged 9–36 months was 44%, 11%, and 25%, respectively. Common predictors of stunting and underweight included low birth weight, short maternal stature, lack of maternal exposure to print media, and maternal consumption of iron–folic acid during pregnancy. A lack of maternal exposure to print media was also associated with wasting. In addition, stunting was linked to male sex and low maternal education, while underweight was associated with children from non-Hindu and non-Muslim religious backgrounds, and maternal lack of autonomy or control over household finances. Wasting, however, was associated with the 24–36 months age group and maternal gestational diabetes.

**Conclusion:**

A high level of undernutrition was observed in the urban slums of Belagavi, with the prevalence of stunting exceeding the national and state averages. Undernutrition was linked to maternal, child, and household factors, including low birth weight, maternal stature, education, and autonomy.

## Introduction

Malnutrition continues to threaten children's ability to thrive and survive, representing one of the greatest social challenges globally (1, 2). The World Health Assembly and Sustainable Development Goals target ending all forms of malnutrition by 2030, yet the progress remains uneven (3, 4). Undernutrition is particularly alarming in urban India, where 30.1% of children under 5 years are stunted, 18.5% are wasted, and 27.3% are underweight (5). Malnourished children face the highest risk of mortality and morbidity, along with some far-reaching consequences, affecting not only individuals but also societal and economic outcomes (6, 7).

In India, the extent of malnutrition is disproportionately high in urban slums, where children are more vulnerable than their counterparts in non-slum urban areas (8, 9). Low- and middle-income countries (LMICs) have experienced a significant growth in urbanization over the past few decades, leading to the development of slums characterized by overcrowding, unhygienic living conditions, and inadequate access to healthcare (9). Migrants to urban slums are particularly at risk, as they transition from a food-secure environment to precarious urban settings. Specific factors that contribute to poor nutritional status in children include a lack of immunization, early weaning, and dependence on street food, exacerbating undernutrition in this population and highlighting the urgent need for nutrition surveillance in these areas (9, 10).

Despite national efforts to address malnutrition, data from urban slums remains limited. Interventions related to malnutrition have historically focused on rural areas, leaving the unique vulnerabilities of urban slums unexplored. Karnataka, one of India's rapidly urbanizing states, exhibits substantial variation in health outcomes, but the nutritional status and its determinants among children in urban slums remain poorly understood. This study aims to assess the nutritional status and determinants of undernutrition among children aged 9–36 months in the urban slums of Belagavi, Karnataka.

## Methods

### Study context

The analysis presented in this study of the prevalence and determinants is from the data of the parent randomized controlled trial (RCT) conducted in the urban slums of Belagavi (CTRI Reg no: CTRI/2022/06/043002). The protocol for the RCT was approved by the Ethical Committee (Human) for Ph.D. Research, KAHER, Belagavi. Permission was obtained from the District Health Office, Taluka Health Office, and medical officers of the respective slum areas of Belagavi to conduct the study. Informed written consent was obtained from all the mothers as per the guidelines given by the Indian Council of Medical Research (ICMR), India.

### Data collection time frame and eligibility screening for the RCT

The data for the current analysis were collected from March to July 2023. After obtaining a list of slums from the Taluka Health Office, it was determined that the slums in Belagavi fell under six primary health centers (PHCs). Permission to conduct the study was then secured from the respective medical officers of these PHCs. Data collected from all eligible and ineligible children aged 9–36 months at the screening time for the registered RCT were included in the present analysis.

### Sample size estimation

The sample size of the randomized controlled trial was *n* = 360, calculated using a 95% confidence interval and 90% power, considering an attrition of 1.05 and design effect of 1.2. The following formula was used:

$$n=\frac{{\left(Z_{1-(\alpha /2)}+Z_{1-\beta }\right)}^{2}\times (SD_{1}^{2}+SD_{2}^{2})}{{\left({\bar{x}}_{1}-{\bar{x}}_{2}\right)}^{2}}\times \mathrm{Attrition}\times \mathrm{Design}\;\mathrm{Effect}$$

The previous randomized controlled trial showed a mean difference in height of the participants pre- and post-intervention of 14.7 ± 2.1 and 13.8 ± 1.7 cm, in the experimental and control groups, respectively (11, 12).

$$\begin{array}{rl} n & =\frac{{\left(1.96+1.29\right)}^{2}\times (2.1^{2}+1.7^{2})}{{\left(14.7-13.8\right)}^{2}}\times 1.05\times 1.2\\ & =120\;\mathrm{in}\;\mathrm{each}\;\mathrm{group}\\ & =360 \end{array}$$

### Data collection and nutritional assessment methods for RCT screening

Data were collected by a trained researcher at the time of the screening of the children for the randomized controlled trial. Mothers/caregivers were interviewed to collect data on correlates such as child, maternal, and household-level characteristics.

Anthropometric data were collected using standardized procedures. For the final measurement, an average of triplicate measurements was utilized. Weight was measured using an electronic scale with a digital screen to the nearest 0.01 kg (child and parent). Height was measured using an infantometer and a stadiometer (child and parents) to the nearest 0.1 cm. A non-flexible tape was used to measure mid-upper arm circumference and head and chest circumference to the nearest 0.1 cm.

Children were screened for signs of nutritional deficiencies. A child was considered to have signs of anemia if they presented with any one of the following: pale skin, pale eyes, pale tongue, or pale or spoon-shaped nails. Signs of a vitamin A deficiency included whether the child exhibited Bitot's spot, dry eyes, or scarring. A vitamin D deficiency was indicated by knock-knees, bowed legs, a widening of the wrist, Harrison's sulcus, or frontal bossing.

### Variable description

Table 1 describes the independent variables considered for this study.

**Table 1:** Independent variables for analysis.

Variable level	Variable description	Categorization
Child level	Age (months)	9–11, 12–23, or 24–36
	Gender	Male or female
	Birth order	First, second, or equal to or more than three
	Birth weight (g)	Low birth weight (<2,500) or normal or above average (≥2,500)
	Child’s status at birth	Preterm (defined as birth before 37 weeks of gestation) or full term
	DI in the previous 3 months	Yes or no
	RTI in the previous 3 months	Yes or no
	Signs of vitamin D deficiency	Yes or no
	Signs of anemia	Yes or no
	Exclusive breastfeeding (months)	<6, 6, or >6
	Dietary preferences	Vegetarian, non-vegetarian, or ovo-vegetarian
	Protein intake	Adequate or inadequate (defined using the acceptable macro-nutrient distribution range) (13)
	Calcium intake	Adequate or inadequate (categorized using RDA) (13)
	Zinc intake	Adequate or inadequate (categorized using EAR) (13)
Maternal level	Age (years)	≤20 or more than 20
	Education	Illiterate, primary education, secondary education, or higher education (5)
	Height (cm)	<155 or ≥155 (14)
	BMI (kg/m^2^)	Normal (18.5–22.9), underweight (<18.5), or overweight (≥23) (15)
	Birth interval	First child, <24 months, or ≥24 months
	Place of delivery	Public, private, or home delivery
	Mode of delivery	Normal or cesarean
	Occupation	Housewife or working
	Exposure to print media	Yes or no
	Exposure to electronic media	Yes or no
	Maternal Hb (g/dl)	Anemia (<11) or normal (≥11)
	Gestational diabetes mellitus	Yes or no (collected using the mother/child health card)
	Pre-eclampsia	Yes or no (collected using the mother/child health card)
	Iron–folic acid consumption	Yes or no
	ANC visits	More than or equal to four or less than four (collected using the mother/child health card).
	Autonomy	Yes or no
	Control of money	Yes or no
Household level	Religion	Hindu, Muslim, or other religion
	Type of family	Nuclear family (defined as parents residing with their unmarried children) or joint family

DI, diarrheal infection; RTI, respiratory tract infection; RDA, recommended dietary allowance; EAR, estimated average requirement; BMI, body mass index; Hb, haemoglobin; ANC, ante-natal checkup.

The outcome variables included the prevalence of stunting, wasting, and underweight among children. Stunting, wasting, and underweight were defined as height-for-age *Z* score (HAZ), weight-for-height *Z* score (WHZ), and weight-for-age *Z* score (WAZ), respectively, more than 2 standard deviations (SD) below the median of the WHO growth standards (16). These outcomes were assessed using the WHO Anthro software (17).

### Statistical analysis

Categorical data were reported as frequencies and percentages, while continuous variables were summarized using means and SD. The chi-square test was used to assess the significant differences between undernutrition and explanatory variables or covariates. In addition, simple or crude logistic regression was used to examine the association between each covariate and the outcome variable. Eventually, multiple or multivariable logistic regression was used to examine the independent effect of each covariate on child malnutrition. We applied three models for the analysis: Model 1 was adjusted for individual child-level characteristics, Model 2 accounted for both child and maternal-level characteristics, and Model 3 incorporated adjustments for child, maternal, and household-level characteristics. Further, the equation for the multivariable logistic regression model is expressed as

log(π/1−π)=β0+β1X1+β2X2+⋯+βmXm

where *π* is the probability of the occurrence of the event (stunting, wasting, or underweight), *βi* is the regression coefficient associated with the reference group, and *Xi* is the explanatory variables. Stata 16 software was used for all the statistical analyses.

## Results

A total of 420 children living in the urban slums of Belagavi were screened for the main study, with complete data available for 362 children in the present analysis.

### Descriptive analysis

**Figure 1:**
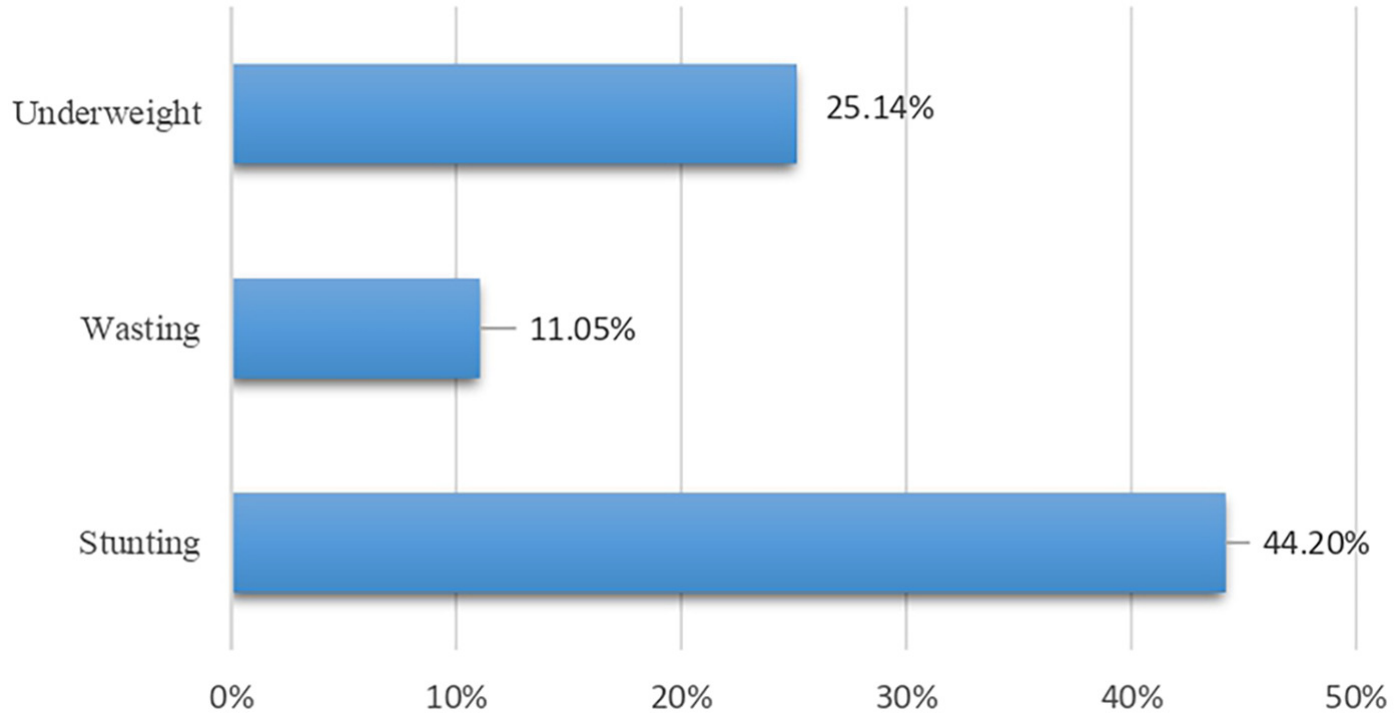
Prevalence of undernutrition in the urban slums of Belagavi among children aged 9–36 months.

As shown in Figure 1, the prevalence among children aged 9–36 months was 44.2% for stunting, 11.05% for wasting, and 25.14% for underweight. Descriptive statistics of the child, maternal, and household-level characteristics are presented in Table 2. The majority of the children were aged 12–36 months (79.5%) and more than half were female (54.1%). Most of the children were first in birth order (44.6%), had normal or above-average birth weight (79.6%), and were full term (80.6%). Diarrhea and respiratory tract infection (RTI) were reported at least once in the previous 3 months in 60.9% and 48.2% of the individuals, respectively. No signs of vitamin A deficiency were observed, while 11.6% showed signs of a vitamin D deficiency and 28.8% exhibited signs of anemia. Nearly all the children had an inadequate intake of calcium (96.7%) and zinc (97.8%) in their diet. The majority of mothers had completed their secondary education (74.6%), were housewives (92.3%), had exposure to electronic media (91.4%), and 74% had no exposure to print media. Most households belonged to the Hindu religion (64.4%) and reported living in a joint family (61.5%).

**Table 2:** Individual, maternal, and household-level characteristics of children.

Variable	Number (%) or mean (standard deviation)
Individual level	
Age (months)	
9–11	74 (20.4)
12–23	146 (40.3)
24–36	142 (39.2)
Sex	
Male	166 (45.9)
Female	196 (54.1)
Birth order	
First	161 (44.6)
Second	138 (38.2)
≥3	62 (17.2)
Birth weight (g)	
Low birth weight (<2,500)	74 (20.4)
Normal or above average (≥2,500)	288 (79.6)
Anthropometric measurements (mean ± SD)	
Height (cm)	78.4 ± 7.99
Weight (kg)	9.73 ± 1.85
Mid-upper arm circumference (cm)	14 ± 1.09
Chest circumference (cm)	45.86 ± 2.64
Head circumference (cm)	45.57 ± 2.02
Child's status at birth	
Full term	290 (80.6)
Preterm	70 (19.4)
Diarrheal infection	
Yes	220 (60.9)
No	141 (39.1)
Respiratory tract infection	
Yes	174 (48.2)
No	187 (51.8)
Signs of vitamin A deficiency	
No	361 (100)
Signs of vitamin D deficiency	
Yes	42 (11.6)
No	319 (88.4)
Signs of anemia	
Yes	104 (28.8)
No	257 (71.2)
Exclusive breastfeeding (months)	
<6	85 (23.6)
6	207 (57.3)
>6	69 (19.1)
Dietary preference	
Vegetarian	63 (17.5)
Non-vegetarian	269 (74.5)
Ovo-vegetarian	29 (8)
Dietary history (mean ± SD)	
Calorie intake	535.2 ± 175.2
Protein intake (g)	14.3 ± 5.4
Calcium intake (mg)	156.1 ± 112.2
Zinc intake (mg)	1.1 ± 0.52
Protein intake	
Adequate	198 (54.6)
Inadequate	165 (45.4)
Calcium intake by RDA	
Adequate	12 (3.3)
Inadequate	351 (96.7)
Zinc intake	
Adequate	8 (2.2)
Inadequate	355 (97.8)
Maternal level	
Age (years)	
≤20	8 (2.2)
>20	353 (97.8)
Education	
Illiterate	10 (2.8)
Primary education	10 (2.8)
Secondary education	270 (74.6)
Higher education	72 (19.9)
Height (cm)	
<155	170 (47.09)
≥155	191 (52.91)
BMI	
Normal (18.5–22.9)	156 (43.2)
Underweight (<18.5)	71 (19.7)
Overweight (≥23)	134 (37.1)
Birth interval	
First child	160 (44.3)
<24 months	26 (7.2)
≥24 months	175 (48.5)
Place of delivery	
Public	169 (46.7)
Private	191 (52.8)
Home	2 (0.6)
Mode of delivery	
Normal	171 (47.4)
Cesarean	190 (52.6)
Occupation	
Housewife	334 (92.3)
Working	28 (7.7)
Exposure to print media	
Yes	94 (26)
No	267 (74)
Exposure to electronic media	
Yes	330 (91.4)
No	31 (8.6)
Maternal history during pregnancy	
Hemoglobin level	
<11 g/dl (anemia)	137 (38.2)
≥11 g/dl (normal)	222 (61.8)
Gestational DM	
Yes	6 (1.7)
No	355 (98.3)
Pre-eclampsia	
Yes	28 (7.8)
No	333 (92.2)
Iron–folic acid consumption	
Yes	319 (88.4)
No	42 (11.6)
ANC	
≥4	237 (65.8)
<4	123 (34.2)
Autonomy	
Yes	265 (73.4)
No	96 (26.6)
Control of money	
Yes	256 (70.9)
No	105 (29.1)
Household level	
Religion	
Hindu	233 (64.4)
Muslim	123 (34)
Other	6 (1.6)
Type of family	
Nuclear	139 (38.5)
Joint	222 (61.5)

LBW, low birth weight; MUAC, mid-upper arm circumference.

Table 3 presents the results of the chi-square tests examining the association between various child, maternal, and household characteristics with different forms of undernutrition (stunting, wasting, and underweight). Stunting was significantly associated with low birth weight, low maternal education, and short maternal height. Wasting was significantly associated with child age. Characteristics including child age, low birth weight, short maternal height, and iron–folic acid (IFA) consumption were significantly associated with underweight.

**Table 3:** Chi-square test results of the association between child, maternal, and household characteristics and undernutrition.

Variable	Stunting		Wasting		Underweight	
	No	Yes	No	Yes	No	Yes
Child level						
Age (months)	*p* = 0.127		*p* = 0.006*		*p* = 0.007*	
9–11	49 (66.2)	25 (33.8)	69 (93.2)	5 (6.8)	65 (87.8)	9 (12.2)
12–23	76 (52.1)	70 (47.9)	136 (93.2)	10 (6.9)	109 (74.7)	37 (25.3)
24–36	78 (54.9)	64 (45.1)	117 (82.4)	25 (17.6)	97 (68.3)	45 (31.7)
Gender	*p* = 0.053		*p* = 0.577		*p* = 0.948	
Male	84 (50.6)	82 (49.4)	146 (87.9)	20 (12.1)	124 (74.7)	42 (25.3)
Female	119 (60.7)	77 (39.3)	176 (89.8)	20 (10.2)	147 (75.0)	49 (25)
Birth order	*p* = 0.371		*p* = 0.922		*p* = 0.208	
First	88 (54.7)	73 (45.3)	143 (88.8)	18 (11.2)	114 (70.8)	47 (29.2)
Second	83 (60.1)	55 (39.9)	122 (88.4)	16 (11.6)	110 (79.7)	28 (20.3)
≥3	31 (50.0)	31 (50.0)	56 (90.3)	6 (9.7)	46 (74.2)	16 (25.8)
Birth weight	*p* = 0.003*		*p* = 0.732		*p* = 0.012*	
LBW	30 (40.5)	44 (59.5)	65 (87.8)	9 (12.2)	47 (63.5)	27 (36.5)
Normal or above average	173 (60.0)	115 (40)	257 (89.2)	31 (10.8)	224 (77.8)	64 (22.2)
Child's status at birth	*p* = 0.644		*p* = 0.109		*p* = 0.878	
Full term	161 (55.5)	129 (44.5)	254 (87.6)	36 (12.4)	217 (74.8)	73 (25.2)
Preterm	41 (58.6)	29 (41.4)	66 (94.3)	4 (5.7)	53 (75.7)	17 (24.3)
Diarrheal infection	*p* = 0.982		*p* = 0.414		*p* = 0.542	
Yes	123 (55.9)	97 (44.1)	198 (90.0)	22 (10)	167 (75.9)	53 (24.1)
No	79 (56.0)	62 (44.0)	123 (87.2)	18 (12.8)	103 (73.1)	38 (26.9)
Respiratory tract infection	*p* = 0.892		*p* = 0.925		*p* = 0.213	
Yes	98 (56.3)	76 (43.7)	155 (89.1)	19 (10.9)	125 (71.8)	49 (28.2)
No	104 (55.6)	83 (44.4)	166 (88.8)	21 (11.2)	145 (77.5)	42 (22.5)
Signs of vitamin D deficiency	*p* = 0.620		*p* = 0.387		*p* = 0.876	
Yes	22 (52.4)	20 (47.6)	39 (98.9)	3 (7.1)	31 (73.8)	11 (26.2)
No	180 (56.4)	139 (43.6)	282 (88.4)	37 (11.6)	239 (74.9)	80 (28.1)
Signs of anemia	*p* = 0.326		*p* = 0.585		*p* = 0.834	
Yes	54 (51.9)	50 (48.1)	91 (87.5)	13 (12.5)	77 (74.0)	27 (26.0)
No	148 (57.6)	109 (42.4)	230 (89.5)	27 (10.5)	193 (75.1)	64 (24.9)
Exclusive breastfeeding (months)	*p* = 0.690		*p* = 0.066		*p* = 0.885	
<6	51 (60.0)	34 (40.0)	76 (89.4)	9 (10.6)	64 (75.3)	21 (24.7)
6	113 (54.5)	94 (45.4)	189 (91.3)	18 (8.7)	156 (75.4)	51 (24.6)
>6	38 (55.1)	31 (44.9)	56 (81.2)	13 (18.8)	50 (72.5)	19 (27.5)
Dietary preference	*p* = 0.745		*p* = 0.886		*p* = 0.270	
Vegetarian	38 (60.3)	25 (39.7)	56 (88.9)	7 (11.1)	51 (80.9)	12 (19.1)
Non-vegetarian	148 (55.0)	121 (45.0)	240 (89.2)	29 (10.8)	200 (74.3)	69 (25.7)
Ovo-vegetarian	16 (55.2)	13 (44.8)	25 (86.2)	4 (13.8)	19 (65.5)	10 (34.5)
Protein intake	*p* = 0.676		*p* = 0.706		*p* = 0.358	
Adequate	113 (57.1)	85 (43.9)	175 (88.4)	23 (11.6)	152 (76.8)	46 (23.2)
Inadequate	90 (54.9)	74 (45.1)	147 (89.6)	17 (10.4)	119 (72.6)	45 (27.4)
Calcium intake	*p* = 0.873		*p* = 0.214		*p* = 0.172	
Adequate	7 (58.3)	5 (41.7)	12 (100.0)	0 (0.0)	11 (91.7)	1 (8.3)
Inadequate	196 (56.0)	154 (44.0)	310 (88.6)	40 (11.4)	260 (74.3)	90 (25.7)
Zinc intake	*p* = 0.726		*p* = 0.313		*p* = 0.415	
Adequate	4 (50.0)	4 (50.0)	8 (100.0)	0 (0.0)	5 (62.5)	3 (37.5)
Inadequate	199 (56.21)	155 (43.8)	314 (88.7)	40 (11.3)	266 (75.1)	88 (24.9)
Maternal level						
Age (years)	*p* = 0.706		*p* = 0.313		*p* = 0.989	
≤20	5 (62.5)	3 (37.5)	8 (100.0)	0 (0.0)	6 (75.0)	2 (25.0)
>20	197 (55.8)	156 (44.2)	313 (88.7)	40 (11.3)	264 (74.8)	89 (25.2)
Education	*p* = 0.022*		*p* = 0.538		*p* = 0.761	
Illiterate	1 (10.0)	9 (90.0)	10 (100.0)	0 (0.0)	7 (70.0)	3 (30.0)
Primary education	7 (70.0)	3 (30.0)	9 (90.0)	1 (10.0)	8 (80.0)	2 (20.0)
Secondary education	153 (56.7)	117 (43.3)	237 (87.8)	33 (12.2)	199 (73.7)	71 (26.3)
Higher education	42 (58.3)	30 (41.7)	66 (91.7)	6 (8.3)	57 (79.2)	15 (20.8)
Height (cm)	*p* = 0.018*		*p* = 0.288		*p* = 0.003*	
<155	84 (49.4)	86 (50.6)	148 (87.1)	22 (12.9)	115 (67.6)	55 (32.4)
≥155	118 (61.8)	73 (38.2)	173 (90.6)	18 (9.4)	155 (81.2)	36 (18.8)
BMI	*p* = 0.467		*p* = 0.633		*p* = 0.942	
Underweight	83 (53.2)	73 (46.8)	139 (89.1)	17 (10.9)	118 (75.6)	38 (24.4)
Normal	44 (62.0)	27 (38.0)	61 (85.9)	10 (14.1)	53 (74.6)	18 (25.4)
Overweight	75 (56.0)	59 (44.0)	121 (90.3)	13 (9.7)	99 (73.9)	35 (26.1)
Birth interval	*p* = 0.721		*p* = 0.991		*p* = 0.252	
First child	88 (55.0)	72 (45.0)	142 (88.7)	18 (11.3)	113 (70.6)	47 (29.4)
<24 months	13 (50.0)	13 (50.0)	23 (88.5)	3 (11.5)	21 (80.8)	5 (19.2)
≥24 months	101 (57.7)	74 (42.3)	156 (89.1)	19 (10.9)	136 (77.7)	39 (22.3)
Place of delivery	*p* = 0.059		*p* = 0.937		*p* = 0.724	
Public	84 (49.7)	85 (50.3)	150 (88.8)	19 (11.2)	124 (73.4)	45 (26.6)
Private	117 (61.3)	74 (38.7)	170 (89.0)	21 (11)	145 (75.9)	46 (24.1)
Home	1 (100.0)	0 (0.0)	1 (100.0)	0 (0.0)	1 (100.0)	0 (0.0)
Mode of delivery	*p* = 0.0781		*p* = 0.513		*p* = 0.646	
Normal	97 (56.7)	74 (43.3)	154 (90.1)	17 (9.9)	126 (73.7)	45 (26.3)
Cesarean	105 (55.3)	85 (44.7)	167 (87.9)	23 (12.1)	144 (75.8)	46 (24.2)
Occupation	*p* = 0.781		*p* = 0.570		*p* = 0.986	
Housewife	188 (56.3)	146 (43.7)	298 (89.2)	36 (10.8)	250 (74.9)	84 (25.1)
Working	15 (53.6)	13 (46.4)	24 (85.7)	4 (14.3)	21 (75)	7 (25)
Exposure to print media	*p* = 0.074		*p* = 0.192		*p* = 0.116	
Yes	60 (63.8)	34 (36.2)	87 (92.5)	7 (7.5)	76 (80.9)	18 (19.1)
No	142 (53.2)	125 (46.8)	234.(87.6)	33 (12.4)	194 (72.7)	73 (27.3)
Exposure to electronic media	*p* = 0.610		*p* = 0.795		*p* = 0.432	
Yes	186 (56.4)	144 (43.6)	293 (88.8)	37 (11.2)	245 (74.2)	85 (25.8)
No	16 (51.6)	15 (48.4)	28 (90.3)	3 (9.7)	25 (80.6)	6 (19.4)
Autonomy	*p* = 0.584		*p* = 0.078		*p* = 0.546	
Yes	146 (55.1)	119 (44.9)	231 (87.2)	34 (12.8)	196 (74)	69 (26)
No	56 (58.3)	40 (41.7)	90 (93.7)	6 (6.3)	74 (77.1)	22 (22.9)
Control of money	*p* = 0.771		*p* = 0.546		*p* = 0.346	
Yes	142 (55.5)	114 (44.5)	226 (88.3)	30 (11.7)	195 (76.2)	61 (23.8)
No	60 (57.1)	45 (42.9)	95 (90.5)	10 (9.5)	75 (71.4)	30 (28.6)
Maternal history during pregnancy						
Hb (g/dl)	*p* = 0.471		*p* = 0.8		*p* = 0.36	
<11	80 (58.4)	57 (41.6)	121 (88.3)	16 (11.7)	99 (72.3)	38 (27.7)
≥11	121 (54.5)	101 (45.5)	198 (89.2)	24 (10.8)	170 (76.6)	52 (23.4)
Gestational diabetes mellitus	*p* = 0.260		*p* = 0.080		*p* = 0.644	
Yes	2 (33.3)	4 (66.7)	4 (66.7)	2 (33.3)	4 (66.7)	2 (33.3)
No	200 (56.3)	155 (43.7)	317 (89.3)	38 (10.7)	266 (74.9)	89 (25.1)
Pre-eclampsia	*p* = 0.597		*p* = 0.489		*p* = 0.351	
Yes	17 (60.7)	11 (39.3)	26 (98.9)	2 (7.1)	23 (82.1)	5 (17.9)
No	185 (55.6)	148 (44.4)	295 (88.6)	38 (11.4)	247 (74.2)	86 (25.8)
Iron–folic acid consumption	*p* = 0.069		*p* = 0.387		*p* = 0.013*	
Yes	173 (54.2)	146 (45.8)	282 (88.4)	37 (11.6)	232 (72.7)	87 (27.3)
No	29 (69.0)	13 (31)	39 (98.9)	3 (7.1)	38 (90.4)	4 (9.5)
ANC visits	*p* = 0.997		*p* = 0.409		*p* = 0.780	
≥4	133 (56.1)	104 (43.9)	213 (89.9)	24 (10.1)	176 (74.3)	61 (25.7)
<4	69 (56.1)	54 (43.9)	107 (87.0)	16 (13.0)	93 (75.6)	30 (24.4)
Household level						
Religion	*p* = 0.063		*p* = 0.891		*p* = 0.055	
Hindu	138 (59.2)	95 (40.8)	208 (89.3)	25 (10.7)	183 (78.5)	50 (21.5)
Muslim	64 (52.0)	59 (48.0)	109 (88.6)	14 (11.4)	85 (69.1)	38 (30.9)
Other	1 (16.7)	5 (83.3)	5 (83.3)	1 (16.7)	3 (50.0)	3 (50.0)
Type of family	*p* = 0.140		*p* = 0.408		*p* = 0.992	
Nuclear	71 (51.1)	68 (48.9)	126 (90.6)	13 (9.4)	104 (74.8)	35 (25.2)
Joint	131 (59.0)	91 (40.1)	195 (87.8)	27 (12.2)	166 (74.8)	56 (25.2)

*Significant at the level *p* < 0.05.

### Association between child, maternal, and household characteristics and undernutrition: crude and multivariable analyses

#### Stunting

Table 4 presents the unadjusted crude and adjusted odds ratios (AORs) for the association between stunting and various child, maternal, and household-level characteristics.

**Table 4:** Determinants of stunting among children in the urban slums.

Independent variable	Crude OR	95% CI	Model I		Model II		Model III	
			AOR	95%	AOR	95%	AOR	95%
Age (months)								
9–11 (reference)								
12–23	1.81*	1.01–3.23	1.75	0.95–3.24	1.75	0.89–3.41	1.75	0.89–3.48
24–36	1.61	0.90–2.88	1.48	0.78–2.80	1.72	0.86–3.47	1.81	0.89–3.67
Sex								
Male (reference)								
Female	0.66	0.44–1.01	0.63*	0.40–0.99	0.6*	0.36–0.99	0.61	0.37–1.01
Birth order								
First (reference)								
Second	0.8	0.50–1.27	0.88	0.54–1.43	1.04	0.07–15.80	1.49	0.11–21.01
≥3	1.21	0.67–2.17	1.28	0.69–2.37	1.43	0.09–22.63	1.64	0.11–23.74
Birth weight								
LBW (reference)								
Normal or above average	0.45**	0.27–0.76	0.44**	0.25–0.75	0.37**	0.20–0.69	0.34***	0.19–0.66
Child's status at birth								
Full term (reference)								
Preterm	0.88	0.52–1.50	0.75	0.42–1.33	0.79	0.42–1.50	0.75	0.39–1.45
Diarrheal infection								
Yes (reference)								
No	0.99	0.65–1.52	1.06	0.68–1.66	0.98	0.58–1.63	0.95	0.57–1.61
Respiratory tract infection								
Yes (reference)								
No	1.03	0.68–1.56	1.08	0.69–1.69	1.19	0.71–1.98	1.23	0.73–2.09
Signs of vitamin D deficiency								
Yes (reference)								
No	0.85	0.45–1.62	0.93	0.46–1.84	1.01	0.47–2.17	1	0.46–2.17
Signs of anemia								
Yes (reference)								
No	0.8	0.50–1.26	0.82	0.50–1.32	0.94	0.55–1.61	0.97	0.56–1.66
Exclusive breastfeeding (months)								
<6 (reference)								
6	1.25	0.75–2.08	1.28	0.73–2.24	1.25	0.67–2.32	1.31	0.69–2.50
>6	1.22	0.64–2.33	1.13	0.56–2.26	1.09	0.51–2.33	1.11	0.51–2.39
Dietary preferences								
Vegetarian (reference)								
Non-vegetarian	1.24	0.71–2.17	1.04	0.57–1.90	0.94	0.49–1.80	0.88	0.46–1.71
Ovo-vegetarian	1.24	0.51–3.00	1.08	0.42–2.80	0.73	0.26–2.05	0.57	0.20–1.67
Protein intake								
Adequate (reference)								
Inadequate	1.09	0.72–1.66	1.02	0.65–1.59	0.91	0.55–1.48	0.84	0.51–1.40
Calcium intake								
Adequate (reference)								
Inadequate	1.1	0.34–3.53	0.63	0.17–2.34	0.43	0.10–1.95	0.42	0.09–1.94
Zinc intake								
Adequate (reference)								
Inadequate	0.78	0.19–3.16	0.67	0.13–3.44	0.42	0.07–2.42	0.38	0.06–2.32
Maternal level								
Age (years)								
≤20 (reference)								
>20	1.32	0.31–5.61			2.8	0.39–20.18	2.72	0.36–20.80
Education								
Illiterate (reference)								
Primary education	0.05*	0.00–0.56			0.05*	0.00–0.65	0.03*	0.00–0.48
Secondary education	0.08*	0.01–0.68			0.06*	0.01–0.58	0.06*	0.01–0.57
Higher education	0.08*	0.01–0.66			0.07*	0.01–0.69	0.07*	0.01–0.74
Height (cm)								
Short (<155) (reference)								
Normal (≥155)	0.60*	0.40–0.92			0.56*	0.34–0.92	0.53*	0.31–0.88
Birth interval								
First child (reference)								
<24 months	1.22	0.53–2.80			1.34	0.08–24.03	0.81	0.05–13.56
≥24 months	0.9	0.58–1.38			0.78	0.05–11.78	0.54	0.04–7.65
BMI								
Normal (reference)								
Underweight	0.7	0.39–1.24			0.67	0.34–1.34	0.63	0.32–1.27
Overweight	0.89	0.56–1.42			0.85	0.49–1.46	0.77	0.44–1.35
Place of delivery								
Public (reference)								
Private	0.625*	0.41–0.95			0.56*	0.34–0.93	0.55*	0.33–0.92
Home								
Mode of delivery								
Normal (reference)								
Cesarean	1.06	0.70–1.61			1.13	0.69–1.87	1.11	0.67–1.84
Occupation								
Housewife (reference)								
Working	1.12	0.51–2.42			0.92	0.38–2.25	1.05	0.42–2.64
Exposure to print media								
Yes (reference)								
No	1.55	0.96–2.52			1.85*	1.03–3.30	1.77	0.97–3.25
Exposure to electronic media								
Yes (reference)								
No	1.21	0.58–2.53			1.04	0.44–2.48	0.92	0.37–2.26
Autonomy								
Yes (reference)								
No	0.88	0.55–1.41			0.73	0.28–1.91	0.69	0.25–1.87
Control of money								
Yes (reference)								
No	0.93	0.59–1.48			1.09	0.43–2.72	1.11	0.42–2.91
Maternal history during pregnancy								
Hb								
<11 (reference)								
≥11	1.17	0.76–1.80			1.04	0.63–1.73	1.02	0.61–1.72
Gestational diabetes mellitus								
Yes (reference)								
No	0.39	0.07–2.14			0.32	0.05–1.98	0.31	0.05–1.93
Pre-eclampsia								
Yes (reference)								
No	1.24	0.56–2.72			1.03	0.39–2.70	0.99	0.38–2.61
Iron–folic acid consumption								
Yes (reference)								
No	0.53	0.27–1.06			0.34*	0.15–0.79	0.38*	0.16–0.88
ANC visits								
≥4 (reference)								
<4	1	0.65–1.55			1.06	0.62–1.84	1.14	0.65–2.00
Household level								
Religion								
Hindu (reference)								
Muslim	1.34	0.86–2.08					1.42	0.79–2.56
Other	7.26	0.84–63.16					11.62	1.00–134.80
Type of family								
Nuclear (reference)								
Joint	0.73	0.47–1.11					0.7	0.42–1.18

* *p* < 0.05 (statistically significant).

** *p* < 0.01 (highly significant).

*** *p* < 0.001 (very highly significant).

Unadjusted estimates showed that children aged 12–23 months had a significantly higher risk of stunting (crude OR: 1.81, 95% CI: 1.01–3.23), but this association lost its significance after adjusting for other factors. Interestingly, sex was not significantly associated with stunting in the bivariate analysis, but in the adjusted model, being female was associated with a lower risk of stunting (AOR: 0.60, 95% CI: 0.36–0.99).

Children with normal or above-average birth weight consistently had a lower risk of stunting, as shown by both the crude (crude OR: 0.45, 95% CI: 0.27–0.76) and adjusted odds ratios (model 3—AOR: 0.34, 95% CI: 0.19–0.66). Regarding maternal characteristics, children of mothers with higher or secondary education were significantly less likely to be stunted (AOR: 0.06–0.07, 95% CI: 0.01–0.74), compared to mothers who were illiterate. Similarly, children of mothers with normal height were less likely to be stunted (AOR: 0.53, 95% CI: 0.31–0.88) compared to those with shorter mothers. Children delivered in private hospitals had a 45% lower risk of being stunted. A unique finding was the influence of maternal exposure to print media: in the adjusted models, children of mothers who lacked such exposure were more likely to be stunted (AOR: 1.85, 95% CI: 1.03–3.30), though this was not observed in the crude estimates. In addition, children of mothers who did not consume IFA tablets during pregnancy paradoxically showed a lower risk of stunting (AOR: 0.38, 95% CI: 0.16–0.88). This was a result that only became significant in the adjusted analyses, warranting further exploration.

#### Wasting

Table 5 highlights the factors associated with wasting. Children aged 24–36 months had a significantly higher risk of wasting in all the models, with an AOR of 4.04 (95% CI: 1.19–13.64). Among maternal characteristics, a lack of exposure to print media was associated with an increased risk of wasting in children (AOR: 3.18, 95% CI: 1.05–9.59). In addition, children of mothers without gestational diabetes had a lower risk of being wasted (AOR: 0.06, 95% CI: 0.01–0.52). Furthermore, the adjusted model indicates that children of mothers lacking autonomy had a decreased risk of being wasted (AOR: 0.1, 95% CI: 0.02–0.50).

**Table 5:** Determinants of wasting among children in urban slums.

Independent variable	Crude OR	95% CI	Model I		Model II		Model III	
			AOR	95%	AOR	95%	AOR	95%
Age (months)								
9–11 (reference)								
12–23	1.01	0.33–3.08	1.08	0.34–3.43	1.13	0.33–3.91	0.95	0.26–3.41
24–36	2.95*	1.08–8.06	3.07*	1.04–9.11	4.12*	1.25–13.56	4.04*	1.19–13.64
Sex								
Male (reference)								
Female	0.83	0.43–1.60	0.85	0.43–1.71	0.71	0.31–1.60	0.7	0.31–1.61
Birth order								
First (reference)								
Second	1.04	0.51–2.13	1.16	0.55–2.46	3.3	0.02–590.38	2.82	0.03–260.59
≥3	0.85	0.32–2.25	1.01	0.36–2.83	3.44	0.02–654.46	2.75	0.03–264.51
Birth weight								
LBW (reference)								
Normal or above average	0.87	0.40–1.92	0.72	0.31–1.69	0.72	0.28–1.85	0.63	0.24–1.68
Child's status at birth								
Full term (reference)								
Preterm	0.43	0.15–1.24	0.43	0.14–1.32	0.55	0.16–1.88	0.49	0.14–1.76
Diarrheal infection								
Yes (reference)								
No	1.32	0.68–2.55	1.35	0.67–2.71	1.49	0.66–3.37	1.64	0.71–3.76
Respiratory tract infection								
Yes (reference)								
No	1.03	0.53–1.99	0.96	0.47–1.96	1.19	0.51–2.73	1.53	0.63–3.75
Signs of vitamin D deficiency								
Yes (reference)								
No	1.71	0.50–5.80	1.7	0.47–6.16	1.92	0.46–8.12	1.69	0.40–7.21
Signs of anemia								
Yes (reference)								
No	0.82	0.41–1.66	0.92	0.44–1.95	0.92	0.40–2.14	0.91	0.39–2.12
Exclusive breastfeeding (months)								
<6 (reference)								
6	0.8	0.35–1.87	0.68	0.28–1.67	0.8	0.29–2.22	0.7	0.25–1.99
>6	1.96	0.78–4.91	1.32	0.49–3.56	1.81	0.59–5.57	1.74	0.57–5.30
Dietary preferences								
Vegetarian (reference)								
Non-vegetarian	0.97	0.40–2.32	0.63	0.24–1.64	0.57	0.20–1.58	0.47	0.16–1.36
Ovo-vegetarian	1.28	0.34–4.77	1.07	0.26–4.36	0.72	0.14–3.66	0.55	0.10–3.01
Protein intake								
Adequate (reference)								
Inadequate	0.88	0.45–1.71	0.92	0.46–1.87	0.55	0.24–1.24	0.5	0.21–1.17
Calcium intake								
Adequate (reference)								
Inadequate			—	—	—	—	—	—
Zinc intake			—	—	—	—	—	—
Adequate (reference)								
Inadequate								
Maternal level								
Literacy status								
Illiterate (reference)								
Primary education	1.22	0.13–11.35			1.89	0.13–27.41	1.6	0.11–23.53
Secondary education	1.53	0.62–3.81			1.85	0.60–5.75	1.66	0.51–5.36
Higher education								
Height (cm)								
<155 (reference)								
≥155	0.7	0.36–1.35			0.57	0.25–1.33	0.53	0.22–1.26
Birth interval								
First child (reference)								
<24 months	1.03	0.28–3.77			0.32	0.00–73.13	0.31	0.00–38.57
≥24 months	0.96	0.49–1.90			0.28	0.00–50.15	0.36	0.00–33.16
BMI								
Normal (reference)								
Underweight	1.34	0.58–3.10			1.76	0.64–4.84	1.84	0.66–5.13
Overweight	0.88	0.41–1.88			0.72	0.30–1.76	0.65	0.26–1.62
Place of delivery								
Public (reference)								
Private	0.97	0.50–1.88			0.86	0.40–1.85	0.82	0.37–1.80
Home								
Mode of delivery								
Normal (reference)								
Cesarean	1.25	0.64–2.42			1.26	0.58–2.75	1.28	0.58–2.83
Occupation								
Housewife (reference)								
Working	1.38	0.45–4.20			0.77	0.19–3.10	0.81	0.20–3.35
Exposure to print media								
Yes (reference)								
No	1.38	0.45–4.20			3.04*	1.05–8.84	3.18*	1.05–9.59
Exposure to electronic media								
Yes (reference)								
No	0.85	0.25–2.93			0.74	0.16–3.37	0.8	0.16–3.93
Autonomy								
Yes (reference)								
No	0.45	0.18–1.12			0.11**	0.02–0.57	0.1**	0.02–0.50
Control of money								
Yes (reference)								
No	0.79	0.37–1.69			2.59	0.67–10.08	2.28	0.59–8.82
Maternal history during pregnancy								
Maternal Hb								
<11 (reference)								
≥11	0.86	0.44–1.69			0.95	0.43–2.11	0.92	0.41–2.07
Gestational diabetes mellitus								
Yes (reference)								
No	0.24	0.04–1.35			0.09*	0.01–0.71	0.06*	0.01–0.52
Pre-eclampsia								
Yes (reference)								
No	1.67	0.38–7.34			1.75	0.30–10.39	1.63	0.27–9.86
Iron–folic acid consumption								
Yes (reference)								
No	0.59	0.17–1.99			0.52	0.12–2.32	0.6	0.14–2.70
ANC								
≥4 (reference)								
<4	1.33	0.68–2.60			1.83	0.75–4.46	1.95	0.78–4.87
Household level								
Religion								
Hindu (reference)								
Muslim	1.07	0.53–2.14					1.9	0.70–5.09
Other	1.66	0.19–14.82					5.78	0.33–102.48
Type of family								
Nuclear (reference)								
Joint	1.34	0.67–2.70					1.66	0.69–3.98

* *p* < 0.05 (statistically significant).

** *p* < 0.01 (highly significant).

*** *p* < 0.001 (very highly significant).

#### Underweight

Table 6 shows the results for the factors associated with underweight. Estimates obtained from the final model (model 4) show that the children aged 12–23 and 24–36 months had a significantly higher risk of being underweight (95% CI: 1.82–12.39). Children with normal or above-average birth weight had a lower risk of being underweight (AOR: 0.43, 95% CI: 0.22–0.86) compared to those with low birth weight. Maternal characteristics of normal height and exposure to print media were protective against underweight (AOR: 0.35, 95% CI: 0.19–0.66; and AOR: 2.16, 95% CI: 1.07–4.38, respectively). Children of mothers who did not consume iron–folic acid tablets during pregnancy had a lower risk of being underweight (AOR: 0.29, 95% CI: 0.09–0.94). However, mother's autonomy was associated with an increased risk of the child being underweight (AOR: 0.20, 95% CI: 0.06–0.66), whereas a maternal lack of control over money led to a fourfold increased risk for the child to be underweight (AOR: 4.05, 95% CI: 1.34–12.25). Interestingly, children from non-Hindu and non-Muslim religious backgrounds had a dramatically increased risk of underweight (AOR: 10.79, 95% CI: 1.23–94.74), though this association was not apparent in the unadjusted analyses.

**Table 6:** Determinants of underweight among children in the urban slums.

Independent variable	Crude OR	95% CI	Model I		Model II		Model III	
			AOR	95%	AOR	95%	AOR	95%
Age (months)								
9–11 (reference)								
12–23	2.45*	1.11–5.40	2.33*	1.02–5.35	2.7*	1.08–6.69	2.7*	1.05–6.92
24–36	3.35**	1.53–7.32	3.21**	1.38–7.46	4.37**	1.73–11.09	4.75**	1.82–12.39
Sex								
Male (reference)								
Female	0.98	0.61–1.58	1.07	0.64–1.79	0.98	0.55–1.74	1.02	0.57–1.84
Birth order								
First (reference)								
Second	0.62	0.36–1.06	0.69	0.39–1.22	1.97	0.08–50.95	2.02	0.10–42.34
≥3	0.84	0.43–1.64	0.89	0.44–1.80	3.07	0.11–83.37	2.37	0.11–51.86
Birth weight								
LBW (reference)								
Normal or above average	0.5*	0.29–0.86	0.49*	0.27–0.88	0.48*	0.25–0.94	0.43*	0.22–0.86
Child's status at birth								
Full term (reference)								
Preterm	0.95	0.52–1.75	0.9	0.46–1.74	1.07	0.51–2.24	1	0.47–2.11
Diarrheal infection								
Yes (reference)								
No	1.16	0.72–1.89	1.17	0.70–1.95	0.99	0.55–1.77	0.96	0.53–1.73
Respiratory tract infection								
Yes (reference)								
No	0.74	0.46–1.19	0.64	0.38–1.08	0.65	0.36–1.16	0.71	0.38–1.30
Signs of vitamin D deficiency								
Yes (reference)								
No	0.94	0.45–1.96	0.99	0.45–2.20	1.23	0.48–3.11	1.18	0.46–3.03
Signs of anemia								
Yes (reference)								
No	0.95	0.56–1.59	0.94	0.54–1.65	0.95	0.51–1.77	0.95	0.51–1.78
Exclusive breastfeeding (months)								
<6 (reference)								
6	1	0.55–1.79	0.89	0.47–1.69	1.15	0.56–2.34	1.11	0.53–2.33
>6	1.16	0.56–2.39	0.93	0.42–2.04	1.14	0.48–2.68	1.09	0.46–2.61
Dietary preference								
Vegetarian (reference)								
Non-vegetarian	1.47	0.74–2.91	1.05	0.50–2.19	0.99	0.44–2.22	0.85	0.37–1.95
Ovo-vegetarian	2.24	0.83–6.02	2.14	0.74–6.19	1.2	0.36–3.97	0.85	0.25–2.95
Protein intake								
Adequate (reference)								
Inadequate	1.25	0.78–2.01	1.2	0.73–2.00	0.98	0.56–1.72	0.89	0.50–1.60
Calcium intake								
Adequate (reference)								
Inadequate	3.81	0.48–29.91	3.23	0.37–28.02	3.63	0.34–38.64	3.73	0.34–41.51
Zinc intake								
Adequate (reference)								
Inadequate	0.55	0.13–2.35	0.62	0.12–3.21	0.43	0.07–2.87	0.38	0.06–2.64
Maternal level								
Age (years)								
≤20 (reference)								
>20	1.01	0.20–5.10			0.55	0.07–4.24	0.61	0.08–4.89
Education								
Illiterate (reference)								
Primary education	0.58	0.07–4.56			0.78	0.08–7.57	0.58	0.05–6.30
Secondary education	0.83	0.21–3.31			0.92	0.19–4.44	0.89	0.18–4.37
Higher education	0.61	0.14–2.66			0.78	0.14–4.44	0.89	0.15–5.19
Height (cm)								
<155 (reference)								
≥155	0.49**	0.30–0.79			0.39**	0.22–0.72	0.35**	0.19–0.66
Birth interval								
First child (reference)								
<24 months	0.57	0.20–1.61			0.26	0.01–8.52	0.18	0.01–5.10
≥24 months	0.69	0.42–1.13			0.32	0.01–8.24	0.31	0.01–6.64
BMI								
Normal (reference)								
Underweight	1.05	0.55–2.02			1.47	0.68–3.18	1.45	0.66–3.19
Overweight	1.1	0.65–1.87			1.19	0.64–2.24	1.07	0.57–2.04
Place of delivery								
Public (reference)								
Private	0.87	0.54–1.41			0.79	0.45–1.38	0.73	0.41–1.30
Home								
Mode of delivery								
Normal (reference)								
Caesarian	0.89	0.56–1.44			0.86	0.49–1.53	0.83	0.47–1.49
Occupation								
Housewife (reference)								
Working	0.99	0.41–2.42			0.76	0.28–2.06	0.86	0.31–2.38
Exposure to print media								
Yes (reference)								
No	1.59	0.89–2.84			2.16*	1.07–4.38	2.04	0.97–4.29
Exposure to electronic media								
Yes (reference)								
No	0.69	0.27–1.74			0.6	0.20–1.76	0.54	0.17–1.69
Autonomy								
Yes (reference)								
No	0.84	0.49–1.46			0.22*	0.07–0.70	0.2**	0.06–0.66
Control of money								
Yes (reference)								
No	1.28	0.77–2.13			3.94*	1.32–11.74	4.05*	1.34–12.25
Maternal history during pregnancy								
Hb								
<11 (reference)								
≥11	0.8	0.49–1.30			0.8	0.45–1.43	0.76	0.42–1.37
Gestational diabetes mellitus								
Yes (reference)								
No	0.67	0.12–3.72			0.46	0.07–3.21	0.37	0.06–2.56
Pre-eclampsia								
Yes (reference)								
No	1.6	0.59–4.34			1.25	0.38–4.09	1.21	0.36–4.10
Iron–folic acid consumption								
Yes (reference)								
No	0.28*	0.10–0.81			0.26*	0.08–0.84	0.29*	0.09–0.94
ANC								
≥4 (reference)								
<4	0.93	0.56–1.54			0.94	0.50–1.79	1.05	0.54–2.04
Household level								
Religion								
Hindu (reference)								
Muslim	1.64	1.00–2.68					1.98	0.99–3.99
Other	3.66	0.72–18.69					10.79*	1.23–94.74
Type of family								
Nuclear (reference)								
Joint	1	0.62–1.63					0.86	0.47–1.57

* *p* < 0.05 (statistically significant).

** *p* < 0.01 (highly significant).

*** *p* < 0.001 (very highly significant).

## Discussion

The article aimed to determine the prevalence of stunting, wasting, and underweight among an understudied population of children aged 9–36 months residing in the urban slums of Belagavi, Karnataka, and examined the key determinants of undernutrition (18–21). The findings revealed a high prevalence of stunting compared to the national estimates and state averages of Karnataka, while the prevalence of wasting and underweight was lower (5). These disparities may reflect an increased availability and accessibility of calorie-dense foods to prevent underweight and wasting, but the overall quality of foods remains poor, leading to stunting (22, 23). Previous studies in the literature report similar findings in urban slums, reflecting the unique environmental and economic conditions present in slums (24–29).

Table 7 summarizes all statistically significant determinants of undernutrition after adjusting for child, maternal, and household-level characteristics, along with their corresponding odds ratios.

**Table 7:** Determinants of undernutrition after adjusting for child, maternal, and household-level characteristics.

Variable	Adjusted odds ratio (Model III)	
	AOR	95% CI
Stunting		
Birth weight		
LBW (reference)		
Normal or above average	0.34***	0.19–0.66
Education		
Illiterate (reference)		
Primary education	0.03*	0.00–0.48
Secondary education	0.06*	0.01–0.57
Higher education	0.07*	0.01–0.74
Height (cm)		
Short (<155) (reference)		
Normal (≥155)	0.53*	0.31–0.88
Place of delivery		
Public (reference)		
Private	0.55*	0.33–0.92
Home		
Iron–folic acid consumption		
Yes (reference)		
No	0.38*	0.16–0.88
Wasting		
Age (months)		
9–11 (reference)		
12–23	0.95	0.26–3.41
24–36	4.04*	1.19–13.64
Exposure to print media		
Yes (reference)		
No	3.18*	1.05–9.59
Autonomy		
Yes (reference)		
No	0.1**	0.02–0.50
Gestational diabetes mellitus		
Yes (reference)		
No	0.06*	0.01–0.52
Underweight		
Age (months)		
9–11 (reference)		
12–23	2.7*	1.05–6.92
24–36	4.75**	1.82–12.39
Birth weight		
LBW (reference)		
Normal or above average	0.43*	0.22–0.86
Maternal height (cm)		
<155 (reference)		
≥155	0.35**	0.19–0.66
Autonomy		
Yes (reference)		
No	0.2**	0.06–0.66
Control of money		
Yes (reference)		
No	4.05*	1.34–12.25
Iron–folic acid consumption		
Yes (reference)		
No	0.29*	0.09–0.94
Religion		
Hindu (reference)		
Muslim	1.98	0.99–3.99
Other	10.79*	1.23–94.74

* *p* < 0.05 (statistically significant).

** *p* < 0.01 (highly significant).

*** *p* < 0.001 (very highly significant).

Key risk factors of stunting identified in the study included being male, aged 12–23 months, low birth weight, having an illiterate mother, shorter maternal stature, and maternal IFA consumption, while delivery in the private health sector emerged as a protective factor. The association with male children aligns with previous studies in the literature (29–32). This could be attributed to male children’s higher metabolic requirements and increased susceptibility to infections, leading to increased nutrient deficits that manifest as stunting. Moreover, children aged 12–23 months are at a critical stage of growth and development, having started weaning with increased physical activity, which places them at an increased risk of stunting, with established findings on growth faltering during this phase (33–35). Low birth weight emerged as a significant risk factor for stunting, emphasizing the importance of interventions targeting maternal health during the early phase of pregnancy (36, 37). The strong association between maternal education and stunting is consistent with a substantial body of literature (29, 38, 39). Maternal education is linked with improved health-seeking behavior, better child-caring practices, and utilization of healthcare services. Educated girls marry at an older age, bear a child later in life, have fewer children, and are better equipped with knowledge and resources to prevent malnutrition (29). Maternal stature was also identified as a key risk factor for stunting and is in line with available literature. Short maternal stature reflects suboptimal nutrition intake during their own childhood and adolescent phase and is linked with a higher risk of intra-uterine growth restriction, further adding to the intragenerational cycle of malnutrition (25, 40–42). Although our findings suggest that delivery in private facilities is associated with lower risk of stunting, this likely reflects underlying socioeconomic advantages rather than differences in care quality. Families choosing private care often have better access to resources, improving postnatal nutrition and childcare. Public facilities cater to more vulnerable populations and may be constrained by high patient loads. Additionally, the observed difference may be influenced by the relatively small sample size, which could limit the generalizability of the results. These findings highlight the need to strengthen postnatal support in the public sector.

In terms of wasting, a marker of the current nutritional status of the child (39), this study found that children aged 24–36 months were at increased risk of wasting, which is in contrast with previous studies that suggest an increased risk at an earlier age (43, 44). This observation of increased risk at an older age could reflect the effect of inadequate nutrition or repeated infections during this crucial phase, leading to wasting.

A history of gestational diabetes was associated with an increased risk of wasting, supporting the concept of “fetal programming,” wherein early exposure to certain adverse conditions can influence a child’s growth and lead to poor health outcomes later in life (45–47). Exposure to print media was found to be a protective factor, consistent with previous research showing that mothers with media exposure are better informed, and this can encourage adequate child-caring practices (47–49).

Underweight was significantly associated with low birth weight and older age, which is well documented in the literature (47, 50, 51). Among maternal risk factors were short maternal stature, IFA consumption, and maternal autonomy. Short maternal stature has been linked with increased risk of underweight in children in previous studies (52). In addition, this study revealed that children from non-Hindu and non-Muslim religions had an increased risk of being underweight, which is in contrast with findings from National Family Health Survey-4 (NFHS-4) data, where Hindu children had a higher risk of being underweight (53).

Protective factors against the child being underweight included maternal exposure to print media and control of money. Maternal exposure to media has been associated with a decreased risk of the child being underweight (19). Maternal control of money is one of the important dimensions of maternal autonomy and has been previously reported to be associated with better nutritional outcomes in children (54).

Contrary to expectation, maternal autonomy did not emerge as a protective factor for wasting or underweight. It is possible that the current study's definition of autonomy was not sufficiently elaborative to understand maternal autonomy (20, 54, 55). The association between IFA consumption and underweight, as with stunting, might reflect low adherence to IFA consumption, misreporting, or some confounding variables (56).

Table 8 presents the key determinants of undernutrition among young children residing in the urban slums of Belagavi city.

**Table 8:** Determinants of undernutrition in the urban slums of Belagavi among children aged 9–36 months

Undernutrition	Determinants
Stunting	Birth weight, maternal education, maternal height, place of delivery, and maternal IFA consumption
Wasting	Child’s age, mother’s exposure to print media, maternal autonomy, and gestational diabetes mellitus
Underweight	Child’s age, birth weight, maternal height, maternal autonomy, maternal control of money, maternal IFA consumption, and religion

## Strengths and limitations

The limitations of the present study include issues related to measurements of independent variables, such as maternal autonomy, which could have been captured with more detailed questions. The study's sample included children aged 9–36 months, whereas collecting data on all children under 5 years, along with a detailed history of past illnesses, would have increased the study’s comprehensiveness. In addition, maternal self-reporting on iron–folic acid use may have introduced recall bias. Moreover, including more household/environmental level characteristics could have provided deeper insights into the correlates of malnutrition.

## Conclusion

The study underscores the persistent burden of undernutrition, particularly stunting, among young children in the urban slums of Belagavi, Karnataka. Key risk factors include individual-level characteristics such as male sex, low birth weight, and critical growth phases, such as at 12–23 months; maternal factors such as shorter stature, low education levels, and limited autonomy; and belonging to a non-Hindu or non-Muslim religion, a household-level determinant. Protective factors, including delivery in private health facilities and maternal exposure to print media, highlight actionable areas for interventions. Empowering mothers through education and increased autonomy is crucial for improving the nutritional status of children and reducing the prevalence of malnutrition.

## Data Availability

The raw data supporting the conclusions of this article will be made available by the authors, without undue reservation.

## References

[1] World Health Organization. Levels and trends in child malnutrition: UNICEF/WHO/World Bank Group joint child malnutrition estimates: key findings of the 2023 edition. Available at: https://www.who.int/publications-detail-redirect/9789240073791 (Accessed February 21, 2024).

[2] Arora A. UNICEF DATA. 2020. Global Nutrition Report 2020. Available at: https://data.unicef.org/resources/global-nutrition-report-2020/ (Accessed July 27, 2024).

[3] The Lancet Child & Adolescent Health. Child malnutrition: hungry for action. Lancet Child Adolesc Health. (2021) 5(7):459. 10.1016/S2352-4642(21)00170-X 34143960

[4] World Health Organization. SDG Target 2.2 Malnutrition. Available at: https://www.who.int/data/gho/data/themes/topics/sdg-target-2_2-malnutrition (Accessed February 22, 2024).

[5] data.gov. Open Government Data (OGD) Platform India (2022). Available at: https://data.gov.in (Accessed February 22, 2024).

[6] Siddiqui F Salam RA Lassi ZS Das JK. The intertwined relationship between malnutrition and poverty. Front Public Health. (2020) 8:453. 10.3389/fpubh.2020.00453 32984245 PMC7485412

[7] Victora CG Christian P Vidaletti LP Gatica-Domínguez G Menon P Black RE. Revisiting maternal and child undernutrition in low-income and middle-income countries: variable progress towards an unfinished agenda. Lancet. (2021) 397(10282):1388–99. 10.1016/S0140-6736(21)00394-9 33691094 PMC7613170

[8] Goudet S Jayaraman A Chanani S Osrin D Devleesschauwer B Bogin B Cost effectiveness of a community based prevention and treatment of acute malnutrition programme in Mumbai slums, India. PLoS One. (2018) 13(11):e0205688. 10.1371/journal.pone.0205688 30412636 PMC6226164

[9] Ezeh A Oyebode O Satterthwaite D Chen YF Ndugwa R Sartori J The history, geography, and sociology of slums and the health problems of people who live in slums. Lancet. (2017) 389(10068):547–58. 10.1016/S0140-6736(16)31650-6 27760703

[10] Roy M. Under-five malnutrition in Indian slums. J Dr NTR Univ Health Sci. (2017) 6:270–1. 10.4103/JDRNTRUHS.JDRNTRUHS_60_17

[11] Gera T Shah D Sachdev HS. Zinc supplementation for promoting growth in children under 5 years of age in low- and middle-income countries: a systematic review. Indian Pediatr. (2019) 56(5):391–406. 10.1007/s13312-019-1537-z 30898990

[12] Mathur NB Agarwal DK. Zinc supplementation in preterm neonates and neurological development: a randomized controlled trial. Indian Pediatr. (2015) 52(11):951–5. 10.1007/s13312-015-0751-6 26615342

[13] Radhika MS Swetha B Kumar BN Krishna NB Laxmaiah A. Dietary and nondietary determinants of nutritional status among adolescent girls and adult women in India. Ann N Y Acad Sci. (2018) 1416(1):5–17. 10.1111/nyas.13599

[14] Kozuki N Katz J Lee ACC Vogel JP Silveira MF Sania A Short maternal stature increases risk of small-for-gestational-age and preterm births in low- and middle-income countries: individual participant data meta-analysis and population attributable fraction. J Nutr. (2015) 145(11):2542–50. 10.3945/jn.115.216374 26423738 PMC6457093

[15] World Health Organization. Regional Office for the Western Pacific. The Asia-Pacific Perspective : Redefining Obesity and Its Treatment. Sydney: Health Communications Australia (2000). Available at: https://iris.who.int/handle/10665/206936 (Accessed August 29, 2024).

[16] de Onis M Onyango A Borghi E Siyam A Blössner M Lutter C. Worldwide implementation of the WHO Child Growth Standards | Public Health Nutrition | Cambridge Core. Available at: https://www.cambridge.org/core/journals/public-health-nutrition/article/worldwide-implementation-of-the-who-child-growth-standards/F413605BFAB559159B434E029598BE58 (Accessed January 7, 2025). 10.1017/S136898001200105X 22717390

[17] World Health Organization. WHO Anthro Survey Analyser and other tools. Available at: https://www.who.int/tools/child-growth-standards/software (Accessed October 17, 2024).

[18] United Nations International Children’s Emergency Fund (UNICEF). UNICEF Conceptual Framework on Maternal and Child Malnutrition. New York: UNICEF. Available at: https://www.unicef.org/reports/state-worlds-children-2019 (Accessed September 10, 2024).

[19] Ahsan KZ Arifeen SE Al-Mamun MA Khan SH Chakraborty N. Effects of individual, household and community characteristics on child nutritional status in the slums of urban Bangladesh. Arch Public Health. (2017) 75:9. 10.1186/s13690-017-0176-x 28239459 PMC5317048

[20] Arulampalam W Bhaskar A Srivastava N. Measuring maternal autonomy and its effect on child nutrition in rural India. Economica. (2024) 91(363):719–39. 10.1111/ecca.12518

[21] Yani D Rahayuwati L Sari C Komariah M Fauziah S. Family household characteristics and stunting: an update scoping review. Nutrients. (2023) 15:233. 10.3390/nu15010233 36615889 PMC9824547

[22] Auma CI Pradeilles R Ohly H Eymard-Duvernay S Brizendine KA Blankenship J Urban nutrition situation in the slums of three cities in Asia during the COVID-19 pandemic. Matern Child Nutr. (2023):e13543. 10.1111/mcn.13543 37814492 PMC12647976

[23] Iannotti LL Dulience SJL Green J Joseph S François J Anténor ML Linear growth increased in young children in an urban slum of Haiti: a randomized controlled trial of a lipid-based nutrient supplement. Am J Clin Nutr. (2014) 99(1):198–208. 10.3945/ajcn.113.063883 24225356 PMC3862455

[24] Usmani G. Health status in India: a study of urban slum and non-slum population. Nurs Res Pract. (2018) 2(1):09–14.

[25] Huey SL Finkelstein JL Venkatramanan S Udipi SA Ghugre P Thakker V Prevalence and correlates of undernutrition in young children living in urban slums of mumbai, India: a cross sectional study. Front Public Health. (2019) 7:191. 10.3389/fpubh.2019.00191 31355176 PMC6639755

[26] Das S Chanani S Shah More N Osrin D Pantvaidya S Jayaraman A. Determinants of stunting among children under 2 years in urban informal settlements in mumbai, India: evidence from a household census. J Health Popul Nutr. (2020) 39(1):10. 10.1186/s41043-020-00222-x 33246506 PMC7693500

[27] Patel K Rawat R Sindhu M. An epidemiological study of acute malnutrition in children of age 6 months to 5 years in an urban slum of Mumbai, Maharashtra. Available at: https://www.researchgate.net/publication/323413629_An_epidemiological_study_of_acute_malnutrition_in_children_of_age_6_months_to_5_years_in_an_Urban_Slum_of_Mumbai_Maharashtra (Accessed September 1, 2024).

[28] Popat CN Chaudhari AI Mazumdar VS Patel SV. A cross sectional study to measure the prevalence of malnutrition and factors associated with malnutrition among under five children of an urban slum of Vadodara city. Available at: https://www.jrmds.in/abstract/a-cross-sectional-study-to-measure-the-prevalence-of-malnutrition-and-factors-associated-with-malnutrition-among-under-f-1549.html (Accessed September 1, 2024).

[29] Murarkar S Gothankar J Doke P Pore P Lalwani S Dhumale G Prevalence and determinants of undernutrition among under-five children residing in urban slums and rural area, Maharashtra, India: a community-based cross-sectional study. BMC Public Health. (2020) 20(1):1559. 10.1186/s12889-020-09642-0 33066763 PMC7565769

[30] Kimani-Murage EW Muthuri SK Oti SO Mutua MK van de Vijver S Kyobutungi C. Evidence of a double burden of malnutrition in urban poor settings in Nairobi, Kenya. PLoS One. (2015) 10(6):e0129943. 10.1371/journal.pone.0129943 26098561 PMC4476587

[31] Otsuka Y Agestika L Widyarani Sintawardani N Yamauchi T. Risk factors for undernutrition and diarrhea prevalence in an urban slum in Indonesia: focus on water, sanitation, and hygiene. Am J Trop Med Hyg. (2019) 100(3):727–32. 10.4269/ajtmh.18-0063 30693865 PMC6402924

[32] Bork KA Diallo A. Boys are more stunted than girls from early infancy to 3 years of age in rural Senegal. J Nutr. (2017) 147(5):940–7. 10.3945/jn.116.243246 28298540

[33] Akhade KS Sankhe LR Akarte SV. Magnitude of malnutrition among underfive children in urban slums of commercial capital of India and its multifactorial causation: a community-based study. J Fam Med Prim Care. (2019) 8(12):3865–70. 10.4103/jfmpc.jfmpc_829_19 PMC6924247 31879627

[34] Singh J Singh T Lal M Mahajan S Ruchika Padda P. Morbidity profile of under-5 slum dwellers of Amritsar city: a descriptive cross-sectional study. J Fam Med Prim Care. (2021) 10(11):4131–6. 10.4103/jfmpc.jfmpc_110_21 PMC8797137 35136778

[35] Islam MM Sanin KI Mahfuz M Ahmed AMS Mondal D Haque R Risk factors of stunting among children living in an urban slum of Bangladesh: findings of a prospective cohort study. BMC Public Health. (2018) 18(1):197. 10.1186/s12889-018-5101-x 29378556 PMC5789576

[36] Jeyakumar A Babar P Menon P Nair R Jungari S Tamboli A Is infant and young child-feeding (IYCF) a potential double-duty strategy to prevent the double burden of malnutrition among children at the critical age? Evidence of association from urban slums in Pune, Maharashtra, India. PLoS One. (2022) 17(12):e0278152. 10.1371/journal.pone.0278152 36455056 PMC9714859

[37] Upadhyay RP Taneja S Strand TA Sommerfelt H Hysing M Mazumder S Early child stimulation, linear growth and neurodevelopment in low birth weight infants. BMC Pediatr. (2022) 22(1):586. 10.1186/s12887-022-03579-6 36209050 PMC9547474

[38] Roja VR Narayanan P Chandra Sekaran VC MG AK. Living environment and health of under-five children in urban slums of a coastal region in South India. Ghana Med J. (2020) 54(4):238–44. 10.4314/gmj.v54i4.6 33883772 PMC8042806

[39] Singh SK Srivastava S Chauhan S. Inequality in child undernutrition among urban population in India: a decomposition analysis. BMC Public Health. (2020) 20(1):1852. 10.1186/s12889-020-09864-2 33272222 PMC7713021

[40] Porwal A Agarwal PK Ashraf S Acharya R Ramesh S Khan N Association of maternal height and body mass index with nutrition of children under 5 years of age in India: evidence from comprehensive national nutrition survey 2016–18. Asia Pac J Clin Nutr. (2021) 30(4):675–86. 10.6133/apjcn.202112_30(4).0014 34967196

[41] United Nations International Children’s Emergency Fund (UNICEF). Programme guidance on maternal nutrition. New York: UNICEF. Available at: https://www.unicef.org/documents/programme-guidance-maternal-nutrition (Accessed September 20, 2024).

[42] Khaliq A Nambiar S Miller YD Wraith D. Assessing the relationship of maternal short stature with coexisting forms of malnutrition among neonates, infants, and young children of Pakistan. Food Sci Nutr. (2024) 12(4):2634–49. 10.1002/fsn3.3945 38628194 PMC11016414

[43] Harding KL Aguayo VM Webb P. Factors associated with wasting among children under five years old in south Asia: implications for action. PLoS One. (2018) 13(7):e0198749. 10.1371/journal.pone.0198749 29969457 PMC6029776

[44] Hossain MM Abdulla F Rahman A. Prevalence and determinants of wasting of under-5 children in Bangladesh: quantile regression approach. PLoS One. (2022) 17(11):e0278097. 10.1371/journal.pone.0278097 36417416 PMC9683614

[45] Bazer FW Spencer TE Wu G Cudd TA Meininger CJ. Maternal nutrition and fetal development. J Nutr. (2004) 134(9):2169–72. 10.1093/jn/134.9.2169 15333699

[46] Calek E Binder J Palmrich P Eibensteiner F Thajer A Kainz T Effects of intrauterine growth restriction (IUGR) on growth and body composition compared to constitutionally small infants. Nutrients. (2023) 15(19):4158. 10.3390/nu15194158 37836441 PMC10574227

[47] Wali N Agho K Renzaho AM. Hidden hunger and child undernutrition in south Asia: a meta-ethnographic systematic review. Asia Pac J Clin Nutr. (2022) 31(4):713–39. 10.6133/apjcn.202212_31(4).0014 36576289

[48] Dhawan D Pinnamaneni R Viswanath K. Association between mass media exposure and infant and young child feeding practices in India: a cross-sectional study. Sci Rep. (2023) 13(1):19353. 10.1038/s41598-023-46734-4 37935737 PMC10630397

[49] Seth P Jain P. What are the key determinants of child malnutrition in India? Empirical evidence from NFHS-4. Indian Econ J. (2023) 71(4):729–47. 10.1177/00194662221137853

[50] Chowdhury MRK Khan HTA Rashid M Kabir R Islam S Shariful Islam M Differences in risk factors associated with single and multiple concurrent forms of undernutrition (stunting, wasting or underweight) among children under 5 in Bangladesh: a nationally representative cross-sectional study. BMJ Open. (2021) 11(12):e052814. 10.1136/bmjopen-2021-052814 34903543 PMC8672009

[51] Kundu RN Hossain MG Haque MA Mahumud RA Pal M Bharati P. Burden of undernutrition among under-five Bengali children and its determinants: findings from demographic and health surveys of Bangladesh and India. PLoS One. (2024) 19(4):e0301808. 10.1371/journal.pone.0301808 38578746 PMC10997093

[52] Li Z Kim R Vollmer S Subramanian SV. Factors associated with child stunting, wasting, and underweight in 35 low- and middle-income countries. JAMA Netw Open. (2020) 3(4):e203386. 10.1001/jamanetworkopen.2020.3386 32320037 PMC7177203

[53] Banerjee S SubirBiswas Roy S Pal M Hossain MG Bharati P. Nutritional and immunization status of under-five children of India and Bangladesh. BMC Nutr. (2021) 7(1):77. 10.1186/s40795-021-00484-6 34852848 PMC8638544

[54] Paul P Saha R. Is maternal autonomy associated with child nutritional status? Evidence from a cross-sectional study in India. PLoS One. (2022) 17(5):e0268126. 10.1371/journal.pone.0268126 35544582 PMC9094570

[55] Arulampalam W Bhaskar A Srivastava N. Does greater autonomy among women provide the key to better child nutrition? Available at: https://papers.ssrn.com/sol3/papers.cfm?abstract_id=2742569 (Accessed September 12, 2024).

[56] Nisar YB Aguayo VM Billah SM Dibley MJ. Antenatal iron-folic acid supplementation is associated with improved linear growth and reduced risk of stunting or severe stunting in south Asian children less than two years of age: a pooled analysis from seven countries. Nutrients. (2020) 12(9):2632. 10.3390/nu12092632 32872329 PMC7551993

